# BCT score predicts chemotherapy benefit in Asian patients with hormone receptor-positive, HER2-negative, lymph node-negative breast cancer

**DOI:** 10.1371/journal.pone.0207155

**Published:** 2018-11-21

**Authors:** Mi Jeong Kwon, Sae Byul Lee, Jinil Han, Jeong Eon Lee, Jong Won Lee, Gyungyub Gong, Peter D. Beitsch, Seok Jin Nam, Sei Hyun Ahn, Byung-Ho Nam, Young Kee Shin

**Affiliations:** 1 College of Pharmacy, Kyungpook National University, Daegu, Korea; 2 Research Institute of Pharmaceutical Sciences, Kyungpook National University, Daegu, Korea; 3 Division of Breast Surgery, Department of Surgery, University of Ulsan College of Medicine, Asan Medical Center, Seoul, Korea; 4 Gencurix, Inc., Seoul, Korea; 5 Department of Health Sciences and Technology, SAIHST, Sungkyunkwan University, Seoul, Korea; 6 Department of Surgery, Samsung Medical Center, Sungkyunkwan University School of Medicine, Seoul, Korea; 7 Department of Pathology, University of Ulsan College of Medicine, Asan Medical Center, Seoul, Korea; 8 Targeted Medical Education, Allentown, Pennsylvania, United States of America; 9 HERINGS, The Institute of Advanced Clinical and Biomedical Research, Seoul, Korea; 10 Laboratory of Molecular Pathology and Cancer Genomics, College of Pharmacy, Seoul National University, Seoul, Korea; 11 Department of Molecular Medicine and Biopharmaceutical Sciences, Graduate School of Convergence Science and Technology, Seoul National University, Seoul, Korea; University of North Carolina at Chapel Hill School of Medicine, UNITED STATES

## Abstract

The Breast Cancer Test (BCT) score has been validated for its ability to predict the risk of distant metastasis in hormone receptor-positive, human epidermal growth factor receptor 2 (HER2)-negative early breast cancer. This study aimed to examine the value of the BCT score for predicting the benefit of adjuvant chemotherapy for Korean women with hormone receptor-positive, HER2-negative, lymph node-negative breast cancer. The study included 346 patients treated with either hormone therapy alone (*n* = 203) or hormone therapy plus chemotherapy (*n* = 143), and compared patient survival between the two treatment groups. The effect of BCT score on patient survival by treatment group was assessed using Cox proportional hazards models. Based on the results, the BCT score was prognostic for distant metastasis-free survival and breast cancer-specific survival in the hormone therapy alone group. There was no significant difference between the treatment groups in terms of 10-year distant metastasis-free survival in the overall patient population. However, when patients were classified as low risk (*n* = 266) and high risk (*n* = 80) according to the BCT score, addition of adjuvant chemotherapy to hormone therapy for patients classified as BCT high-risk group led to a significant improvement in 10-year distant metastasis-free survival, from 65.4% to 91.9% (hazard ratio, 0.18; 95% confidence interval, 0.05–0.64; *P* = 0.003); in contrast, there was no benefit for the BCT low-risk group. The stratification of patients according to the BCT score also identified clinically high-risk patients who may not benefit from chemotherapy. Results were similar for breast cancer-specific survival. In conclusion, the BCT score was not only of prognostic value but was also a predictor of chemotherapy benefit for Korean patients with hormone receptor-positive, HER2-negative, lymph node-negative breast cancer.

## Introduction

Adjuvant chemotherapy is used to reduce the recurrence risk of hormone receptor-positive, human epidermal growth factor receptor 2 (HER2)-negative early breast cancer; however, most patients with this condition are successfully treated with adjuvant hormone therapy alone without recurrence [1]. The decision to include adjuvant chemotherapy alongside hormone therapy is based on traditional clinicopathological parameters such as tumor size and nodal status [1] or clinical factor-based tools such as Adjuvant! Online [2, 3], which leads to overtreatment with chemotherapy for many patients [4]. Several multigene molecular signatures, including the Oncotype DX 21-gene recurrence score, MammaPrint 70-gene signature, PAM50-based Prosigna risk of recurrence, and EndoPredict, have been developed to better predict recurrence or distant metastasis in patients with hormone receptor-positive early breast cancer [5, 6]. Among prognostic molecular assays, the Oncotype DX 21-gene assay [7–9] and the MammaPrint 70-gene assay [10, 11] were also validated to predict chemotherapy benefit for patients with hormone receptor-positive early breast cancer. However, the predictive value of these assays in Asian patients with breast cancer remains unclear.

In a previous study [12], we developed and validated a novel prognostic model called the Breast Cancer Test (BCT) score to predict the risk of 10-year distant metastasis in patients with hormone receptor-positive, HER2-negative early breast cancer treated with hormone therapy alone. The BCT score was calculated based on measurement of relative expression of six genes (*UBE2C*, *TOP2A*, *RRM2*, *FOXM1*, *MKI67*, and *BTN3A2*) and two clinical variables (nodal status and tumor size). The results demonstrated that the BCT score provides better prognostic information than traditional clinicopathological parameters alone, such as tumor size, lymph node status, and histologic grade. Here, we assessed whether the BCT score can predict the benefit from adjuvant chemotherapy for patients with hormone receptor-positive, HER2-negative, lymph node-negative breast cancer.

## Materials and methods

### Ethical statement and patients

To determine whether the BCT score can predict the benefit of adjuvant chemotherapy in patients with hormone receptor-positive, HER2-negative, lymph node-negative breast cancer, this study included patients in the validation cohort from our previous study, who were treated with either hormone therapy alone or hormone therapy plus chemotherapy [12]. Among 525 patients with lymph node-negative breast cancer in the validation cohort from two medical centers in Korea (Asan Medical Center and Samsung Medical Center, Seoul), 179 patients from Samsung Medical Center were excluded because they consisted of only chemotherapy-treated patients. Finally, 346 patients with hormone receptor-positive (estrogen receptor [ER]-positive or progesterone receptor [PR]-positive), HER2-negative, lymph node-negative breast cancer who were treated with either hormone therapy alone or hormone therapy plus chemotherapy at Asan Medical Center were included in the analysis. Patients had undergone curative resection for primary tumors with lymph node dissection at Asan Medical Center between 1998 and 2006.

As described in detail previously [12], ER and PR status were determined by immunohistochemistry (IHC) [13], and HER2 positivity was defined based on results of IHC or fluorescence in situ hybridization [14]. The decision of whether to use adjuvant chemotherapy was based on clinical judgment. Most of the chemotherapy regimens used comprised cyclophosphamide, methotrexate, and 5-fluorouracil.

This study was approved by the institutional review board of Asan Medical Center and performed in accordance with the Declaration of Helsinki. Because the study was retrospective in nature, the requirement for informed consent was waived. Patient information was anonymized and de-identified prior to analysis.

### Categorization of risk groups

Patients were categorized into BCT high-risk and BCT low-risk groups according to the BCT score criteria used in our previous study [12]. Briefly, patients with a BCT score <4 were classified as low risk, while those with a BCT score ≥4 were classified as high risk. Clinical risk was determined using the modified version of Adjuvant! Online as in the MINDACT (Microarray in Node-Negative Disease May Avoid Chemotherapy) trial [11]. Details regarding clinical risk assessment in this study are provided in S1 Table.

### Statistical analysis

The primary endpoint was distant metastasis-free survival at 10 years. Distant metastasis-free survival was defined as the time from the date of primary tumor resection to the date of detection of distant metastasis. The secondary endpoint was breast cancer-specific survival, which was defined as the time from the date of primary tumor resection to the date of breast cancer-specific death. Univariate and multivariate analyses were performed using the Cox proportional hazards regression models. All hazard ratios were reported with 95% confidence intervals (CIs). The probability of survival was estimated using the Kaplan-Meier method, and statistical differences in survival rates between groups were assessed using the log-rank test. Treatment effect in each BCT risk group was also assessed using the Cox proportional hazards models. Differences were considered statistically significant at *P* < 0.05. All statistical analyses were performed using R 3.2.0 (http://r-project.org).

## Results

### Patient characteristics

A total of 346 patients with hormone receptor-positive, HER2-negative, lymph node-negative breast cancer who were treated with either hormone therapy alone or hormone therapy plus chemotherapy were included in this study. Patient characteristics are provided in Table 1. The median age of the patients was 47.0 years (range, 25.5–80.0), and 15.3% (*n* = 53) were <40 years old. Most patients had ER-positive and PR-positive breast cancers (85.3%, *n* = 295). Based on the seventh edition of the American Joint Committee on Cancer (AJCC) staging system, all patients had stage I (66.8%) or II (33.2%) disease. Among them, 203 were treated with hormone therapy alone (58.7%), and 143 were treated with hormone therapy plus chemotherapy (41.3%). The median follow-up period was 9.46 years (range, 0.67–16.79). Overall, 80 patients (23.1%) were classified as BCT high risk and 266 patients (76.9%) as BCT low risk.

**Table 1:** Characteristics of the study population.

Patients (*n* = 346)	No. of Patients	%
Age (years)		
<40	53	15.32
40–49	156	45.09
≥50	137	39.60
Tumor size (cm)		
≤2	231	66.76
2–5	113	32.66
>5	2	0.58
ER and PR status		
ER-positive/PR-positive	295	85.26
ER-positive/PR-negative	42	12.14
ER-positive/PR-unknown	2	0.58
ER-negative/PR-positive	7	2.02
AJCC stage (7th edition)		
I	231	66.76
II	115	33.24
Histologic grade		
1	46	13.29
2	243	70.23
3	57	16.47
Adjuvant treatment		
Hormone therapy	203	58.67
Hormone therapy + chemotherapy	143	41.33
BCT score		
Low risk (<4)	266	76.88
High risk (≥4)	80	23.12
Clinical risk		
Low risk	208	60.12
High risk	138	39.88

Abbreviations: AJCC, American Joint Committee on Cancer; BCT, Breast Cancer Test; ER, estrogen receptor; PR, progesterone receptor.

ER and PR status were assessed by immunohistochemistry.

Clinical risk was determined using the modified version of Adjuvant! Online as in the MINDACT (Microarray in Node-Negative Disease May Avoid Chemotherapy) trial.

### Prognostic value of the BCT score

**Fig 1:**
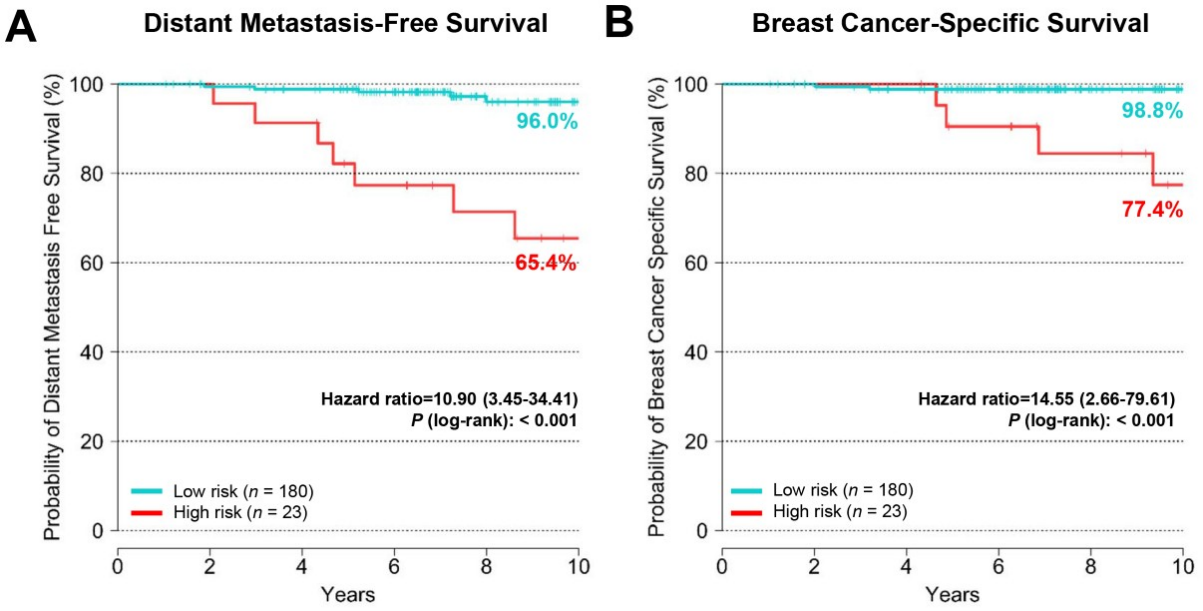
Kaplan-Meier plots for distant metastasis and breast cancer-specific death in patients with hormone therapy alone. (A) Distant metastasis-free survival and (B) breast cancer-specific survival in low-risk (*n* = 180) and high-risk (*n* = 23) groups stratified according to the BCT score. Patients with a BCT score <4 were classified as low risk, while those with a BCT score ≥4 were classified as high risk.

The BCT score was prognostic for distant metastasis-free survival and breast cancer-specific survival within the hormone therapy alone group (Fig 1). The Kaplan-Meier survival analysis showed a significant difference in distant metastasis-free survival between the BCT low-risk and high-risk groups (*P* < 0.001) (Fig 1A). The 10-year distant metastasis-free survival estimates for the BCT low-risk and high-risk groups were 96.0% (95% CI, 92.5–99.7%) and 65.4% (95% CI, 47.3–90.5%), respectively. The BCT high-risk group also had a significantly shorter breast cancer-specific survival than the BCT low-risk group (*P* < 0001) (Fig 1B).

### Benefit of chemotherapy for patient groups classified according to the BCT score

**Fig 2:**
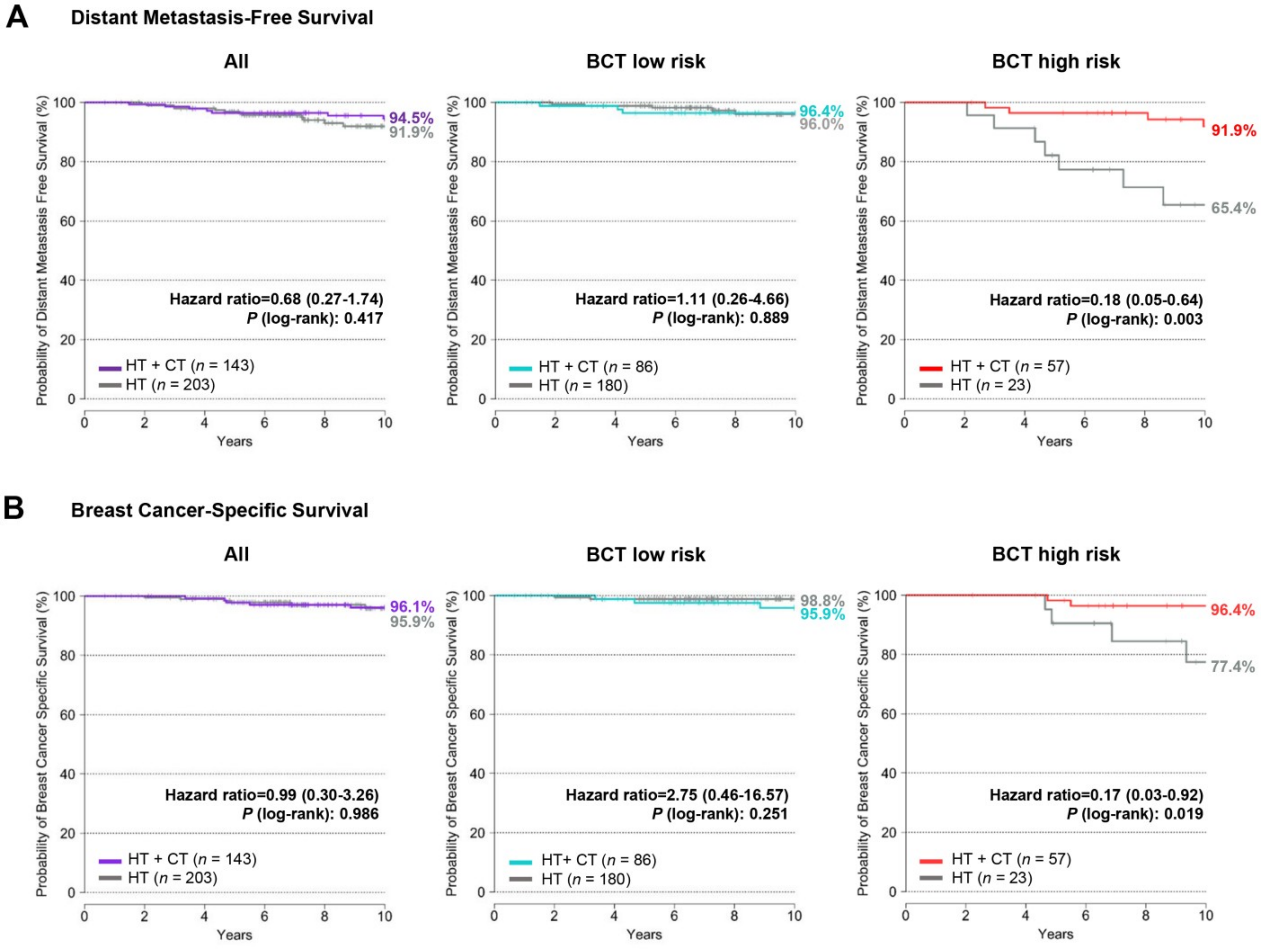
Distant metastasis-free survival and breast cancer-specific survival according to treatment and BCT risk score. (A) Distant metastasis-free survival and (B) breast cancer-specific survival by treatment group within the low-risk (*n* = 266) and high-risk (*n* = 80) groups stratified according to the BCT score. Patients were treated with either hormone therapy alone (HT) or hormone therapy plus chemotherapy (HT+ CT).

There was no significant difference in 10-year distant metastasis-free survival between patients treated with hormone therapy plus chemotherapy and those treated with hormone therapy alone in the entire patient population (*P* = 0.417) (Fig 2A). To assess the predictive value of the BCT score for chemotherapy benefit, we analyzed the chemotherapy benefit in the BCT low-risk and high-risk groups. The Kaplan-Meier analysis showed that the 10-year distant metastasis-free survival estimates for the BCT high-risk group improved significantly from 65.4% to 91.9% after addition of chemotherapy to hormone therapy (hazard ratio, 0.18; 95% CI, 0.05–0.64; *P* = 0.003) (Fig 2A). In contrast, there was no significant difference in 10-year distant metastasis-free survival between the two treatment groups within the BCT low-risk group (*P* = 0.889) (Fig 2A).

Similar results were observed when chemotherapy benefit was analyzed for breast cancer-specific survival (Fig 2B). There was a significant benefit in breast cancer-specific survival from chemotherapy in the BCT high-risk group (hazard ratio, 0.17; 95% CI, 0.03–0.92; *P* = 0.019), but there was no benefit in the BCT low-risk group (*P* = 0.251). For the BCT high-risk group, the 10-year breast cancer-specific survival rates of the hormone therapy plus chemotherapy group and the hormone therapy alone group were 96.4% and 77.4%, respectively.

To evaluate treatment effects according to the BCT risk group, we analyzed the interaction between BCT risk group and chemotherapy treatment. The interaction between chemotherapy treatment and BCT risk group was significant for distant metastasis-free survival (*P* = 0.076) and breast cancer-specific survival (*P* = 0.030).

Among clinical variables, age at the time of surgery and adjuvant treatment (hormone therapy alone *vs*. hormone therapy plus chemotherapy) were significantly associated with risk of distant metastasis for the BCT high-risk group; however, this was not the case for the BCT low-risk group (Table 2). Multivariate analysis revealed that, for the BCT high-risk group, addition of chemotherapy to hormone therapy was an independent factor associated with decreased risk of distant metastasis (hazard ratio, 0.26; 95% CI, 0.07–0.97; *P* = 0.045) (Table 2).

**Table 2:** Effect of clinical factors and adjuvant treatment on prediction of distant metastasis-free survival according to BCT risk group.

	Univariate Analysis				Multivariate Analysis			
	Hazard ratio	95% CI		*P* value	Hazard ratio	95% CI		*P* value
**High-risk group (*n* = 80)**								
Age at surgery	1.08	1.01	1.15	**0.020**	1.05	0.98	1.12	0.138
Size (≤2 cm *vs*. >2 cm)	0.55	0.16	1.88	0.342	-	-	-	-
Histologic grade (Grade 1/2 *vs*. Grade 3)	0.67	0.18	2.54	0.557	-	-	-	-
ER	1.14	0.76	1.71	0.514	-	-	-	-
PR	1.04	0.81	1.34	0.760	-	-	-	-
Adjuvant treatment (hormone therapy *vs*. hormone therapy + chemotherapy)	0.18	0.05	0.64	**0.007**	0.26	0.07	0.97	**0.045**
**Low-risk group (*n* = 266)**								
Age at surgery	0.97	0.90	1.05	0.506	-	-	-	-
Size (≤2 cm *vs*. >2 cm)	0.52	0.06	4.23	0.542	-	-	-	-
Histologic grade (Grade 1/2 vs. Grade 3)	<0.01	0.00	Inf	0.998	-	-	-	-
ER	0.99	0.60	1.62	0.969	-	-	-	-
PR	0.84	0.66	1.07	0.158	-	-	-	-
Adjuvant treatment (hormone therapy vs. hormone therapy + chemotherapy)	1.11	0.26	4.66	0.889	-	-	-	-

Abbreviations: BCT, Breast Cancer Test; CI, confidence interval; ER, estrogen receptor; Inf, infinite; PR, progesterone receptor.

ER and PR status were assessed by immunohistochemistry.

Hazard ratios with *P* values <0.05 are shown in bold.

### Benefit of chemotherapy for patient groups classified according to clinical risk assessment

**Fig 3:**
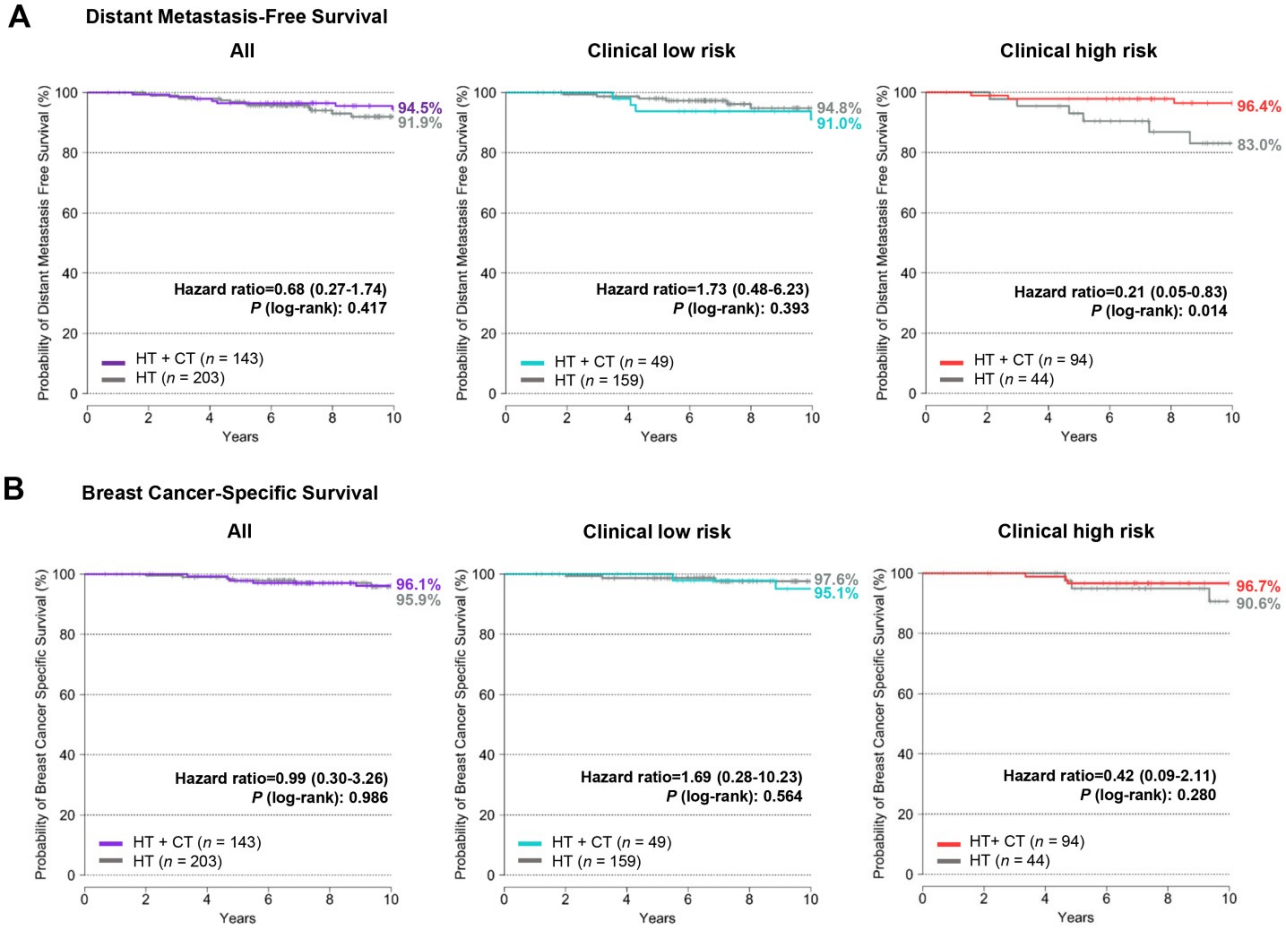
Distant metastasis-free survival and breast cancer-specific survival according to treatment and clinical risk assessment. (A) Distant metastasis-free survival and (B) breast cancer-specific survival by treatment within the low-risk (*n* = 208) and high-risk (*n* = 138) groups stratified according to the modified version of Adjuvant! Online as in the MINDACT (Microarray in Node-Negative Disease May Avoid Chemotherapy) trial. Patients were treated with either hormone therapy alone (HT) or hormone therapy plus chemotherapy (HT + CT).

To compare the predictive value of the BCT score in terms of chemotherapy benefit with that of traditional clinicopathological factors, we performed a clinical risk assessment using the modified version of Adjuvant! Online [11]. A total of 138 patients (39.9%) were categorized into a clinical high-risk group and 208 patients (60.1%) into a clinical low-risk group (Table 1). Similar to the results obtained from BCT risk assessment, adjuvant chemotherapy provided a significant benefit in 10-year distant metastasis-free survival for the clinical high-risk group, but not for the clinical low-risk group (Fig 3A). For the clinical high-risk group, the 10-year distant metastasis-free survival of patients treated with hormone therapy plus chemotherapy (96.4%) was significantly higher than that of patients treated with hormone therapy alone (83.0%) (hazard ratio, 0.21; 95% CI, 0.05–0.83; *P* = 0.014). However, the chemotherapy benefit in 10-year distant metastasis-free survival for the clinical high-risk group (13.4%) was lower than that for the BCT high-risk group (26.5%).

The Kaplan-Meier analysis revealed that the addition of chemotherapy to hormone therapy provided no significant improvement in 10-year breast cancer-specific survival for the clinical high-risk group (*P* = 0.280) (Fig 3B); in contrast, there was a significant improvement in 10-year breast cancer-specific survival for the BCT high-risk group (Fig 2B).

Finally, patients were divided into four risk groups according to the BCT score and modified version of Adjuvant! Online. Among the 346 patients, 260 (75.1%) had concordant results for clinical and BCT risk: 194 patients were BCT low risk/clinical low risk, and 66 were BCT high risk/clinical high risk. Meanwhile, the results for 86 patients (24.9%) were discordant: 72 patients were BCT low risk/clinical high risk, and 14 patients were BCT high risk/clinical low risk. In patients with discordant results who were BCT low risk/clinical high risk, the 10-year distant metastasis-free survival rates of those treated with hormone therapy plus chemotherapy and those treated with hormone therapy alone were 97.7% and 100%, respectively (S1 Fig), with no significant difference (*P =* 0.433). Similar results were observed for breast cancer-specific survival (S2 Fig). These findings indicate that there was no significant benefit from chemotherapy in patients classified as clinical high risk but BCT low risk.

## Discussion

In this study, we found that patients classified as high risk according to the BCT score showed significant improvement in survival after addition of chemotherapy to hormone therapy, whereas there was no significant survival benefit for those at low risk according to the BCT score. Similar to results of the BCT score, patients at high risk according to clinical assessment derived significant benefit from adjuvant chemotherapy. However, the improvement in 10-year distant metastasis-free survival after addition of chemotherapy to hormone therapy was greater for the BCT high-risk group (26.5%) than for the clinical high-risk group (13.4%). Among the 346 patients, 24.9% showed discordant results between the two risk assignment methods. Importantly, for the BCT low-risk/clinical high-risk group, there was no survival benefit after addition of chemotherapy to hormone therapy. These findings indicate that the BCT score may identify patients who will not benefit from chemotherapy among patients classified as clinical high risk.

Previous studies revealed that two multigene expression-based assays, the Oncotype DX 21-gene recurrence score [7–9] and the MammaPrint 70-gene signature [10, 11], can predict chemotherapy benefit for patients with hormone receptor-positive early breast cancer. However, the predictive value of these assays in Asian patients with breast cancer remains unclear because these assays were validated in Western countries. Of note, racial differences in the prognostic value of these assays have been reported. African American women with ER-positive, lymph node-negative breast cancer were more likely to be categorized as high risk according to the 21-gene recurrence score than white women [15], and non-Hispanic black women had significantly higher recurrence scores than non-Hispanic white women [16]. Additionally, a study reported that 70-gene signature results were different between European and Asian patients [17]. Therefore, the predictive value of these assays may depend on the ethnic or racial background of the patient. To our knowledge, the present study is the first to report the predictive value of a multigene assay for adjuvant chemotherapy benefit in an Asian population. In this regard, the BCT score may provide additional information on which to base treatment decisions for Asian patients with breast cancer.

Our results suggest that the BCT risk score may provide better prognostic and predictive value than clinical factors alone and thus may help patients avoid the unnecessary toxic effects of chemotherapy. However, this study has some limitations. The study has a retrospective design, was performed in a single center, and included a limited number of patients. Further well-designed prospective studies are therefore required to demonstrate the predictive utility of the BCT score in making treatment decisions.

## Conclusions

In summary, the BCT score predicted whether a patient with hormone receptor-positive, HER2-negative, lymph node-negative breast cancer will derive significant benefit from addition of adjuvant chemotherapy to hormone therapy. Moreover, the BCT score could identify patients who may not benefit from adjuvant chemotherapy despite being classified as high risk according to clinical assessment. Overall, the results suggest that the BCT score was not only of prognostic value but was also a reliable predictor of chemotherapy benefit for patients with hormone receptor-positive, HER2-negative, lymph node-negative breast cancer.

## Supporting information

S1 Fig Distant metastasis-free survival by treatment in risk groups classified by BCT score and clinical assessment. (A) BCT low-risk/clinical low-risk group, (B) BCT high-risk/clinical high-risk group, (C**)** BCT low-risk/clinical high-risk group, and (D) BCT high-risk/clinical low-risk group. Clinical risk was classified using the modified version of Adjuvant! Online as in the MINDACT (Microarray in Node-Negative Disease May Avoid Chemotherapy) trial. Patients were treated with either hormone therapy (HT) alone or hormone therapy plus chemotherapy (HT + CT).
(TIF)

S2 Fig Breast cancer-specific survival by treatment in risk groups classified by BCT score and clinical assessment. (A) BCT low-risk/clinical low-risk group, (B) BCT high-risk/clinical high-risk group, (C) BCT low-risk/clinical high-risk group, and (D) BCT high-risk/clinical low-risk group. Clinical risk was classified using the modified version of Adjuvant! Online as in the MINDACT (Microarray in Node-Negative Disease May Avoid Chemotherapy) trial. Patients were treated with either hormone therapy (HT) alone or hormone therapy plus chemotherapy (HT + CT).
(TIF)

S1 Table Classification of patients according to clinical risk assessment using the modified version of Adjuvant! Online. (DOC)
