# Supplementary figures and images for: BCT score predicts chemotherapy benefit in Asian patients with hormone receptor-positive, HER2-negative, lymph node-negative breast cancer
Source: PLoS One. 2018 Nov 21;13(11):e0207155. doi: 10.1371/journal.pone.0207155 (PMC6248959; doi:10.1371/journal.pone.0207155)

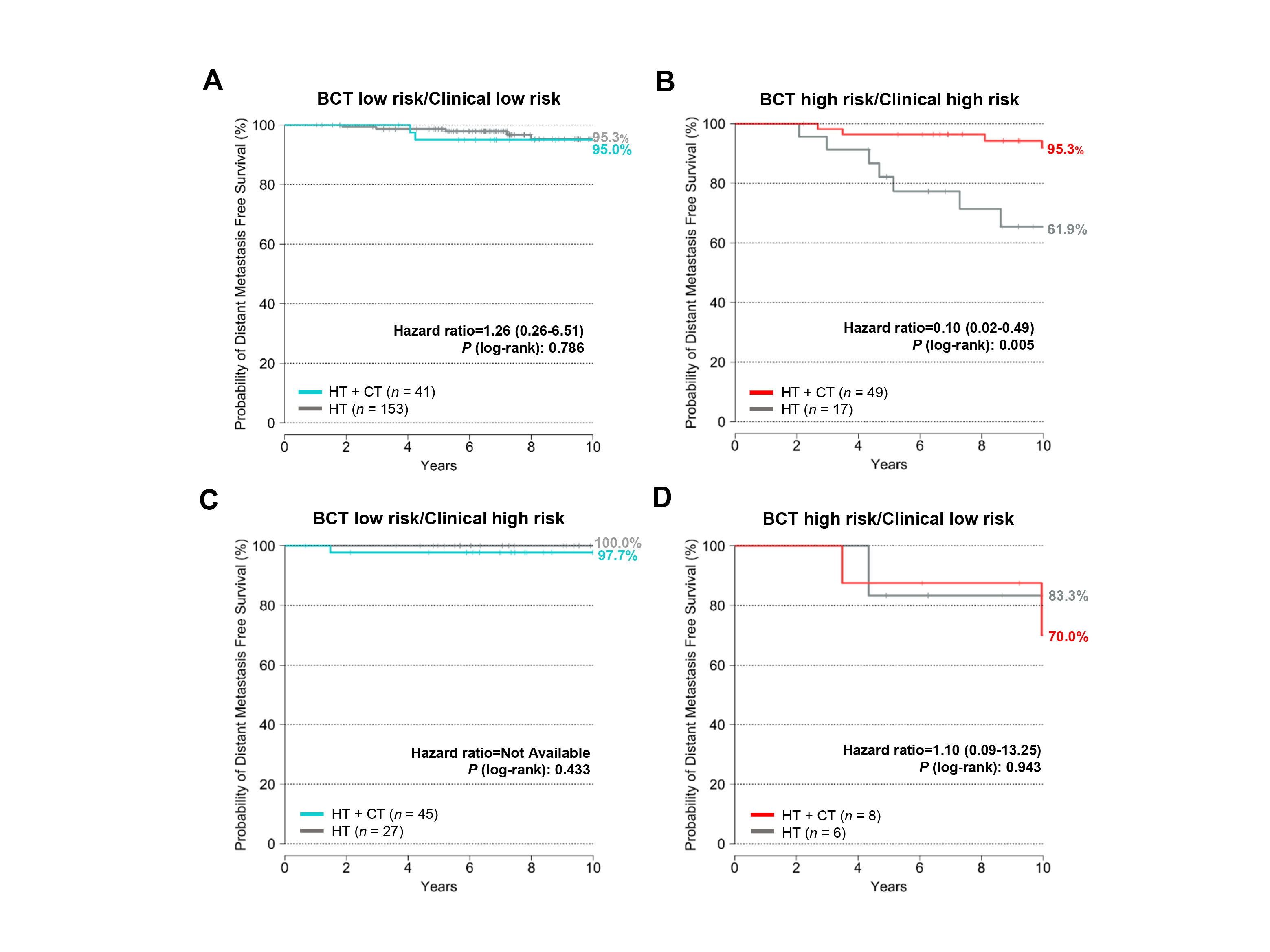

Supplement: S1 Fig — (A) BCT low-risk/clinical low-risk group, (B) BCT high-risk/clinical high-risk group, (C) BCT low-risk/clinical high-risk group, and (D) BCT high-risk/clinical low-risk group. Clinical risk was classified using the modified version of Adjuvant! Online as in the MINDACT (Microarray in Node-Negative Disease May Avoid Chemotherapy) trial. Patients were treated with either hormone therapy (HT) alone or hormone therapy plus chemotherapy (HT + CT). (TIF) [file pone.0207155.s001.tif]

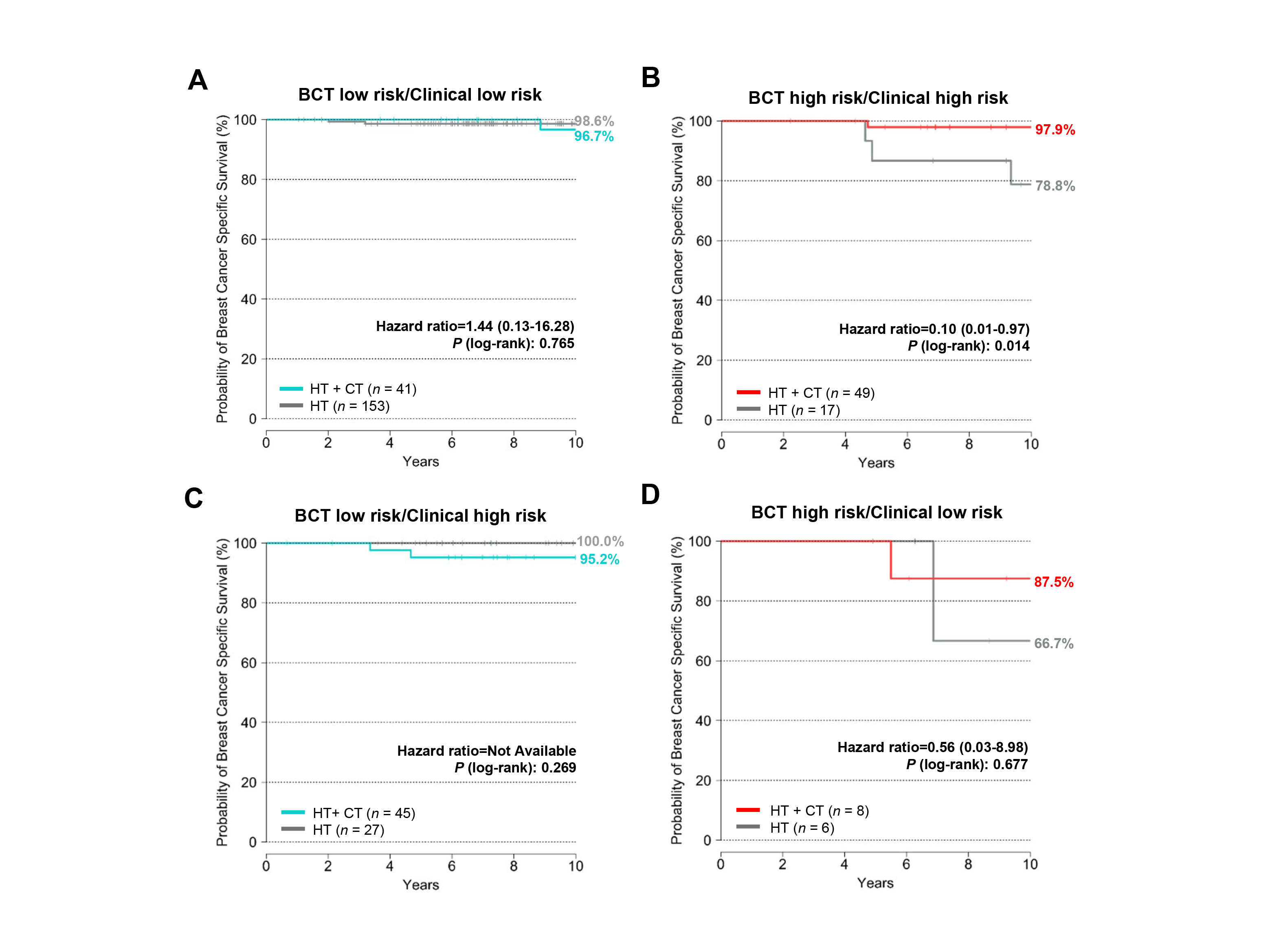

Supplement: S2 Fig — (A) BCT low-risk/clinical low-risk group, (B) BCT high-risk/clinical high-risk group, (C) BCT low-risk/clinical high-risk group, and (D) BCT high-risk/clinical low-risk group. Clinical risk was classified using the modified version of Adjuvant! Online as in the MINDACT (Microarray in Node-Negative Disease May Avoid Chemotherapy) trial. Patients were treated with either hormone therapy (HT) alone or hormone therapy plus chemotherapy (HT + CT). (TIF) [file pone.0207155.s002.tif]
