# Supplementary material for: BCT score predicts chemotherapy benefit in Asian patients with hormone receptor-positive, HER2-negative, lymph node-negative breast cancer
Source: PLoS One. 2018 Nov 21;13(11):e0207155. doi: 10.1371/journal.pone.0207155 (PMC6248959; doi:10.1371/journal.pone.0207155)
Supplement: S1 Table — (DOC) [file pone.0207155.s003.doc]

**S1 Table. Classification of Patients According to Clinical Risk Assessment Using the Modified Version of Adjuvant! Online.**

| **ER status** | **Grade** | **Tumor**  **size** | **Clinical**  **risk** |
| --- | --- | --- | --- |
| Positive | Well differentiated | ≤3 cm | C-low |
|  |  | 3.1-5 cm | C-high |
|  | Moderately differentiated | ≤2 cm | C-low |
|  |  | 2.1-5 cm | C-high |
|  | Poorly differentiated  or undifferentiated | ≤1 cm | C-low |
|  |  | 1.1-5 cm | C-high |
| Negative | Well differentiated | ≤2 cm | C-low |
|  |  | 2.1-5 cm | C-high |
|  | Moderately differentiated  OR  poorly differentiated  or undifferentiated | ≤1 cm | C-low |
|  |  | 1.1-5 cm | C-high |

Abbreviation: ER, estrogen receptor

Clinical risk was determined using the modified version of Adjuvant! Online as in the MINDACT (Microarray in Node-Negative Disease May Avoid Chemotherapy) trial.
